# Advancement of clinical practice in delivering CAR T-cell therapy: impact on healthcare resource utilization and comparison with autologous stem cell transplantation in patients with relapsed/refractory large B-cell lymphomas

**DOI:** 10.1007/s00277-025-06564-y

**Published:** 2025-09-06

**Authors:** Martin Fehr, Matthias Naegele, Michael Greiling

**Affiliations:** 1 Department of Medical Oncology and Haematology, Cantonal Hospital St. Gallen/HOCH Health Ostschweiz, St. Gallen, Switzerland; 2 Institute for Workflow-Management in Health Care, European University of Applied Sciences, Cologne, Germany

**Keywords:** CAR T-cell, ASCT, Large B-cell lymphoma, Healthcare resource utilization

## Abstract

In patients with relapsed/refractory (R/R) large B-cell lymphoma (LBCL) who are either refractory to first-line therapy or relapse within 12 months, chimeric antigen receptor (CAR) T-cell therapy is more effective than salvage chemotherapy followed by high-dose chemotherapy and autologous stem cell transplantation (ASCT) as second-line therapy. Adoption of CAR T-cell therapy into routine clinical practice involves a period of adaptation and refinement of clinical processes. We aimed to document the evolution of clinical processes for CAR T-cell therapy during 2022 and 2023, and compare healthcare resource utilization (HCRU) associated with CAR T-cell and ASCT processes in routine clinical practice. ClipMed^PPM^ software-based process modeling was used to assess HCRU for patients with R/R LBCL receiving CAR T-cell or ASCT therapy, mapping 991 and 1174 processes associated with CAR T-cell therapy in 2023 and 2022, respectively, and 1874 processes associated with ASCT over both years. Improvements in lymphodepletion therapy administration and assessment and management of CAR T-cell therapy-specific adverse events led to a 5-day (30%) reduction in hospitalization and a 15% decrease in total personnel time in the CAR T-cell therapy process from 2022 to 2023. HCRU for CAR T-cell therapy was almost half that of ASCT, with 77% less personnel time for therapy administration. Hospitalization for CAR T-cell therapy was 70%-75% shorter than for ASCT therapy (11–13 vs. 44 days). These patient-centered process efficiencies provide patients with reduced hospitalization time. Understanding this evolution is vital for addressing complexities of advanced treatments, enhancing patient care quality, and optimizing resource allocation.

**Supplementary Information:**

The online version contains supplementary material available at 10.1007/s00277-025-06564-y.

## Introduction

Large B-cell lymphomas (LBCL), with an estimated 150,000 new cases annually worldwide, constitute almost 30% of all cases of B-cell non-Hodgkin’s lymphoma [1]. Diffuse large B-cell lymphoma (DLBCL) is the most frequently occurring aggressive LBCL, with the most common subtype being DLBCL not otherwise specified; other subtypes include primary mediastinal B-cell lymphoma and high-grade B-cell lymphoma [1]. While data on the global incidence of DLBCL are not available, an annual incidence of 7.2 per 100,000 people has been reported in the United States, and this is dependent on age, sex, and ethnicity [2]. The median age at first diagnosis is approximately 70 years [3], and between 50% and 60% of all patients are cured with rituximab-based chemoimmunotherapy in the first-line setting [4]. However, a considerable proportion of patients are refractory to treatment or experience relapse [1].

Before chimeric antigen receptor (CAR) T-cell therapy became available, salvage chemotherapy followed by high-dose chemotherapy and autologous stem cell transplantation (ASCT) was the second-line therapy option for younger patients with chemotherapy-relapsed/refractory (R/R) DLBCL; this therapy carried the highest chance for durable outcome [5]. Due to patients with R/R LBCL often being older and having several comorbidities, only a limited proportion of patients are able to receive ASCT. Additionally, only around half of the patients who are candidates for transplantation respond to the initial salvage therapy and qualify for ASCT, which itself has an overall cure rate of only 25–35% [6]. In the CORAL study, which assessed the efficacy of different salvage regimens followed by high-dose chemotherapy and ASCT in patients with R/R DLBCL [7], the median progression-free survival (PFS) was 7.1 months and the median overall survival (OS) achieved was only 10.0 months. Similarly, SCHOLAR-1, an international, multicohort retrospective study, demonstrated that, for patients with refractory disease or relapse within 12 months, outcomes with ASCT were consistently poor across patient subgroups and study cohorts with DLBCL [8]. More effective therapies are clearly needed to improve outcomes in patients with R/R LBCL.

CAR T-cell therapy provides a new treatment option with the potential to achieve sustained remission in patients with R/R LBCL in addition to R/R follicular lymphoma (FL). Several CAR T-cell products are available in Europe, including tisagenlecleucel (tisa-cel; Kymriah®; approved in 2018) [9], axicabtagene ciloleucel (axi-cel, Yescarta®; approved in 2018) [10], and lisocabtagene maraleucel (liso-cel; Breyanzi®; approved 2022) [11].

The superior efficacy of CAR T-cell therapy versus salvage chemotherapy followed by high-dose chemotherapy and standard of care ASCT as second-line therapy for patients with R/R LBCL has been reported previously. In the phase 3 ZUMA-7 trial at a median follow-up of 47.2 months, 4-year OS was significantly higher with axi-cel than with standard care (54.6% vs. 46.0%; hazard ratio [HR] for death 0.73; 95% confidence interval [CI] 0.54–0.98; *P* = 0.03) [12]. Similarly, in the TRANSFORM study, liso-cel demonstrated significantly higher event-free survival (EFS) compared with standard of care [13, 14]. A deepening of response was noted after a median follow-up of 33.9 months with an EFS of 29.5 months reported with liso-cel compared with 2.4 months for standard of care (HR 0.375; 95% CI 0.259–0.542) and continued improvements in PFS and duration of response [15].

Salvage chemotherapy followed by high-dose chemotherapy and ASCT is a highly standardized therapy with a 20-year history characterized by well-defined protocols and is established in clinical practice. In contrast, CAR T-cell therapy remains a relatively novel approach [9–11] with opportunity for optimization of treatment protocols in routine clinical practice. Our hospital introduced CAR T-cell therapy for B-cell lymphomas in September 2021; its introduction was delayed by the COVID-19 pandemic. From this time and throughout 2022, our center has treated approximately 14 patients overall: 10 for LBCL and 4 for mantle cell lymphoma, and these numbers are increasing. Our current analysis assessed the evolution of the clinical pathway for CAR T-cell therapy from 2022 to 2023 and the impact on associated healthcare resource utilization (HCRU) in patients with R/R LBCL. In addition, by comparing the HCRU for CAR T-cell therapy with salvage chemotherapy followed by high-dose chemotherapy and ASCT, our analysis complements an earlier single-center analysis conducted at University Hospital, Zurich, Switzerland, which demonstrated CAR T-cell therapy to have substantially lower HCRU, with an overall shorter duration of treatment and fewer hospital days for CAR T-cell therapy when compared with salvage chemotherapy followed by high-dose chemotherapy and ASCT [18].

The current study aims to contribute to the growing body of literature surrounding the impact of CAR T-cell therapy on HCRU, while also providing insights for healthcare providers on the integration of novel therapies into routine clinical practice. Understanding this progression of CAR T-cell treatment pathway is critical for several reasons, as it not only informs more optimal resource allocation within healthcare systems but also aligns closely with the principles of value-based healthcare. By directing resource allocation based on informed insights into improved patient care and experience, healthcare providers can ensure the delivery of enhanced quality of care and the judicious use of available resources.

## Methods

### Software-based procedural health economic analysis (SPHA)

The methodology has been reported previously [16]. An SPHA was conducted to determine the HCRU in inpatient and outpatient settings for patients with R/R LBCL who received CAR T-cell therapy in 2022 or 2023, or high-dose chemotherapy and ASCT with or without salvage therapy (henceforth described as ASCT therapy). Platinum-based chemotherapy, most commonly rituximab, ifosfamide, carboplatin, and etoposide (R-ICE) was the chemotherapy of choice for this modeling exercise. The key milestones of the SPHA methodology are described in Table 1.

**Table 1:** The software-based procedural health economic analysis: key milestones

Milestones	Description
**Determination of core competencies and clinical pathway**	Identification of a homogeneous patient group with a specific medical condition, which could be assigned to a specific DRG with a comparable diagnosis, procedures (diagnostics, therapy), and treatment duration. Development of the clinical pathway including all services, such as diagnostics, therapies, supportive services, and other ancillary services based on clinical SOPs and guidelines with the assistance of the responsible HCPs (e.g., physician, nurses, specialized functions).
**Premodeling**	Based on the clinical pathway, modeling of a “draft” process flow, composed of all subprocesses from admission to discharge, using the ClipMed^PPM^ software.
**Main modeling – ** **model validation:** **resources type and unit utilization collection**	Adaptation of the premodeled clinical pathway and process flow. Input and data collection, such as process duration, responsibilities, and probability of execution. Data validation through structured individual and/or workshop settings discussions with physician and nursing staff.
**Controlling and data processing and allocation**	Processing of primary controlling data and calculation, using ClipMed^PPM^ software.
**Quality control and validation**	Quality assurance for completeness, relevance, plausibility, and consistency.

*DRG* diagnosis-related group, *HCP* healthcare professional, *SOP* standard operating procedure

The SPHA involved process-oriented modeling of the clinical pathways examined in this study [17]. The analysis included comprehensive mapping of treatment components and processes, as well as assessments of process duration, the roles and responsibilities of personnel involved, and the probabilities of successful execution at each stage incurred throughout the treatment process [18]. In the scope of this study, the clinical pathway of each therapy, including all services, such as diagnostics, therapies, supportive services, and other ancillary services, was determined based on clinical standard operating procedures (SOPs) at the Cantonal Hospital St. Gallen. Patient selection for therapy was guided by these clinical SOPs, which were based on national reimbursement requirements. In addition, an assessment of every case was performed by the institution’s interdisciplinary tumor board. (For further details, see Supplementary Materials). Guidelines were provided by responsible healthcare professionals (HCPs; e.g., physicians, nurses, specialized functions). The clinical pathway was defined for a homogeneous patient population with a specific medical condition assigned to a specific diagnosis-related group (DRG) with a comparable diagnosis, procedures, and treatment duration. This modeling deliberately excluded transfers to intensive care units (ICUs), the management of severe adverse events (AEs) and other comorbidities unrelated to R/R LBCL, as appropriate when representing a standard patient. By focusing on a homogeneous patient population, the study aimed to represent the average HCRU for standard treatment pathways.

Proprietary ClipMed^PPM^ software was used to model the clinical process flow for all processes, spanning from the first to last doctor-patient contact. The model was then validated using structured individual interview methods and workshop settings with multidisciplinary HCP groups who were involved from patient admission to patient discharge to ensure that the modeled process flow exemplified a standardized, clinically significant, and frequently provided form of care. Subsequently, the collected information, proposed changes, and suggestions were documented and implemented in the modeling software.

Various methods were considered for collecting the execution time of sub-processes, including direct observation, self-recording by staff, the use of flowcharts, time measurement using a stopwatch (Refa method [19]), and estimation based on experience. Considering the high effort involved and the small deviations between the methods, estimation was chosen. In a clinical environment where the staff are highly occupied, estimation provided a practical and efficient solution for determining throughput times. With this rationale, the multidisciplinary HCP team provided data on process duration, measured in minutes, required for the execution of each process step based on their experience. The execution time always referred to the homogeneous patient group defined in the core competence. To ensure accuracy, the estimated times were compared with the average values of all analyses for plausibility. In instances of significant deviations, further reviews with the involved professional groups were conducted for validation. An arithmetic mean was calculated from the comparison of multiple estimates for patients.

Once these data were fully processed and quality checked, verification of the modeling was performed based on the criteria of completeness, relevance, plausibility, and consistency.

### Data sources

The clinical pathway was mapped based on hospital SOPs, clinical guidelines and input from multidisciplinary HCP teams involved from patient admission to patient discharge. The type and quantity of resource utilized in each process step within the treatment pathway was also provided by the multidisciplinary HCP team.

### Time period of study

These data were derived from the Cantonal Hospital St. Gallen, Switzerland. Between January 2022 and December 2022, the ASCT pathway therapy and initial CAR T-cell pathway therapy (referred to as CAR T-cell 2022 therapy) were assessed. Over the course of 2022 and 2023, substantial experience was gained, thereby enhancing the clinical practice for CAR T-cell therapy. Following these adaptations to the CAR T-cell therapy, the updated pathway was assessed from November 2023 to March 2024 (referred to as CAR T-cell 2023 therapy).

### Compliance with ethics guidelines

These data were derived from SOPs and hospital staff expert interviews; individual patient data were not reported.

## Results

### Treatment delivery pathway and processes mapping

**Fig. 1:**
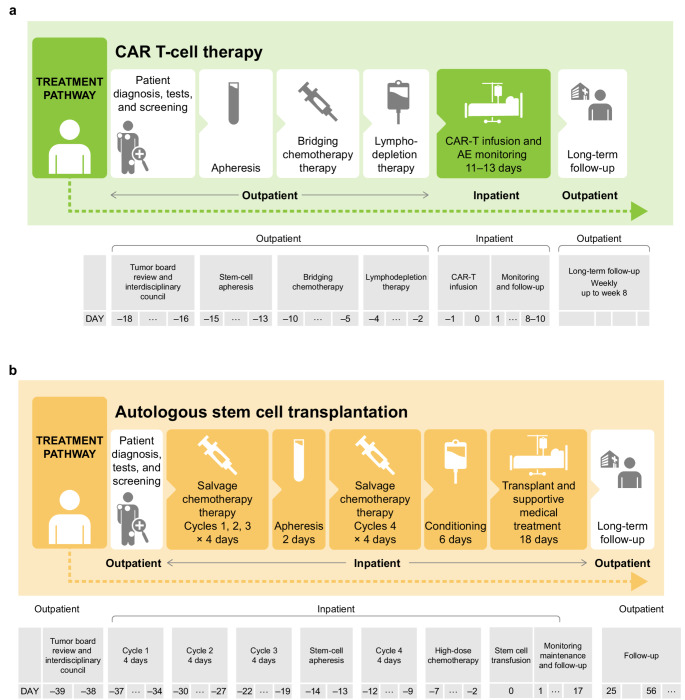
Simplified CAR T-cell 2023 therapy (**a**) and ASCT therapy (**b**) pathways. Days and routine care between treatment components are not included. *ASCT* autologous stem cell transplantation, *CAR* chimeric antigen receptor

**Fig. 2:**
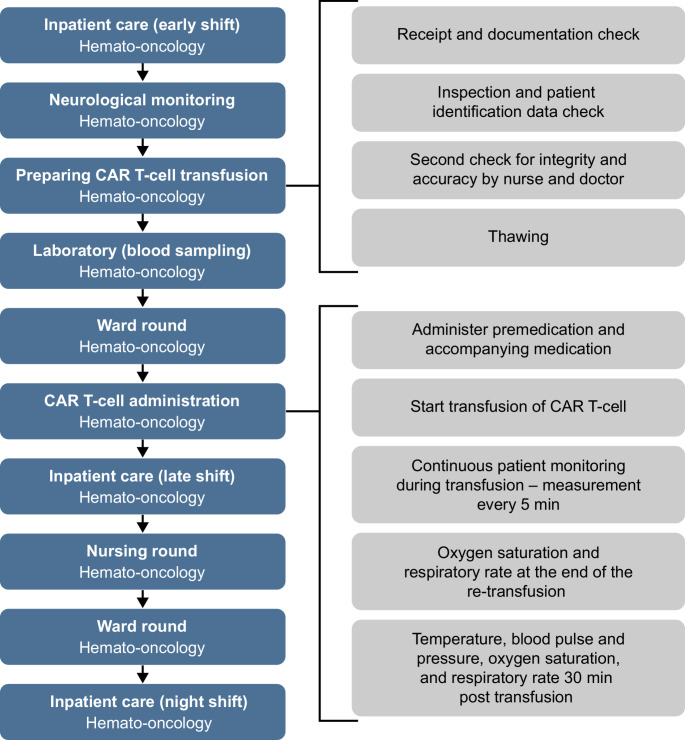
Services and underlaying processes mapping. Example Day “0” re-transfusion of CAR T-cells of CAR T-cell 2023 therapy. Blue blocks depict services provided and grey blocks depict the underlying activities within each service process. *CAR* chimeric antigen receptor

The full clinical pathway for CAR T-cell 2023 therapy shown in Fig. S1 depicts the entire duration of the treatment provision. A simplified version is shown in Fig. 1A. Treatment components and distinct processes for each treatment day were determined, and an example of a day in which re-transfusion of CAR T-cells took place is shown in Fig. 2. The full ASCT pathway is described in Fig. S2. The simplified ASCT pathway is described in Fig. 1B.

Processes (991 for the CAR T-cell procedure and 1874 for the ASCT procedure) were mapped and classified into 5 categories: admission, diagnostics, inpatient services, therapy, and discharge.

Examples of processes involved in the “admission” category include time associated with various administrative tasks, such as scheduling appointments, collecting insurance information, and additional activities performed by medical staff, such as collecting patients’ medical histories and collecting medical reports; planning diagnostic and therapeutic procedures; and ensuring thorough documentation.

The “diagnostics” category covered a range of laboratory tests and imaging techniques. Examples of diagnostic procedures are laboratory tests, electroencephalograms, echocardiograms, electrocardiograms, continuous magnetic resonance tomography, positron emission tomography-computed tomography, pulmonary function tests, and the interpretation of results from these tests.

“Inpatient treatment” mainly encompassed nursing services and physician services. Nursing services included monitoring vital signs, assisting with personal hygiene, administering medications, organizing meals, and providing patient education. Physician services included: conducting rounds; assessing results of laboratory tests and imaging studies and prescribing treatments, with a special focus on detecting and managing CAR T-cell specific toxicities; documenting patient progress; and performing minor procedures.

The “therapy” category involved the preparation and execution of therapeutic procedures and interventions. This included chemotherapy administration, apheresis, cell reinfusion, physical therapy, and the documentation of these procedures. It also included supportive therapies like pain management, nutritional support, and rehabilitation services.

Finally, the “discharge” category consisted of several components. Physician discharge involved performing a final examination, providing discharge summaries, issuing discharge letters, and writing prescriptions. Nursing discharge included giving discharge instructions, documenting follow-up care needs, and educating the patient about recovery. Additionally, this category may involve organizing follow-up appointments and arranging support in the home if required.

The processes were delivered by different functions and further analyzed by HCP departments, such as physician (e.g., hematologists) and nursing services. Functional services may include services from departments, such as cardiology, infectious diseases, neurology, psycho-oncology, and personnel, such as care and support workers. Additional support staff refers to hospital overhead services, including finance, human resources, and cleaners.

### Advancement of CAR T-cell therapy pathway

**Fig. 3:**
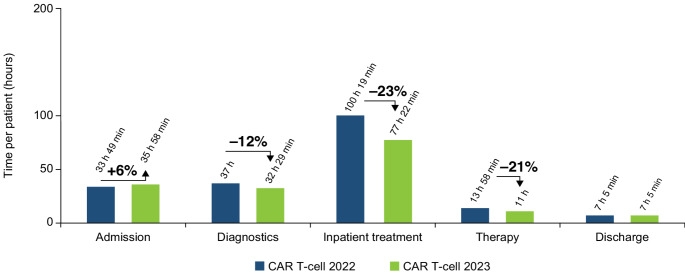
Personnel time by process category in the provision of CAR T-cell 2022 therapy compared with CAR T-cell 2023 therapy. *CAR* chimeric antigen receptor

The primary drivers of the overall adaptation in processes were, firstly, the transition of selected inpatient procedures to the outpatient setting prior to the CAR T-cell infusion phase. This included the administration of lymphodepleting chemotherapy and baseline laboratory tests and imaging assessments. Secondly, in the post-CAR T-cell infusion phase, an internal review determined that the frequency of assessments for CAR T-cell specific toxicities—specifically cytokine release syndrome (CRS) and immune effector cell-associated neurotoxicity syndrome (ICANS)—could be appropriately reduced from 5 to 3 times every 24 h without compromising patient safety. Thirdly, the management of CRS and ICANS further evolved as reflected in the joint publication of best practice guidelines of the relevant professional associations (European Society for Blood and Marrow Transplantation [EBMT]; Joint Accreditation Committee of the International Society for Cellular Therapy and EBMT; European Hematology Association); consequently, the respective institutional SOPs were amended accordingly, resulting in modified administration patterns of specific drugs [20, 21]. This 3-fold approach not only streamlined the patient care process but also enhanced the efficiency of resource utilization while maintaining a strong focus on patient safety and well-being. As a result, there was a notable reduction of the CAR T-cell 2023 therapy pathway compared with the CAR T-cell 2022 therapy pathway. This translated into a 5-day (30%) shorter hospitalization, 22 fewer therapy steps, and reduced underlying processes for the CAR T-cell 2023 therapy pathway by 183 processes (16%) (Table 2). The total personnel time for patient cases and service provisions was reduced by 15% from 192 h 10 min in 2022 to 163 h 53 min in 2023. Inpatient procedure time was reduced by 23% from 100 h 19 min in 2022 to 77 h 22 min in 2023 (Fig. 3).

**Table 2:** Comparison of CAR T-cell therapy provision in 2023 versus 2022

	2023	2022	Change (%)
**Hospitalization days**	11–13	16–18	28–31
**Clinical therapy steps ** **(i.e.**,** clinical services)**	226	248	9
**Underlaying processes**	991	1174	16
**Total personnel time for patient case and services provision**	163 h 53 min	192 h 10 min	15

*CAR* chimeric antigen receptor

**Fig. 4:**
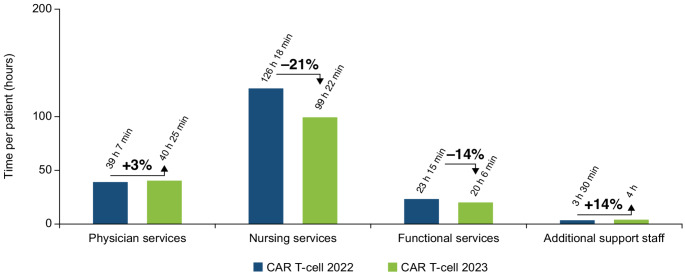
Total time by HCP group in the provision of CAR T-cell 2022 therapy compared with CAR T-cell 2023 therapy. *CAR* chimeric antigen receptor, *HCP* healthcare professional

When comparing the HCRU by HCP group (Fig. 4), the time associated with the physician, functional services, and additional support staff was generally comparable between the CAR T-cell 2022 and 2023 therapies. The main driver of the overall reduction in HCRU by HCPs in 2023 was nursing services, which was reduced by 21% from 126 h 18 min in 2022 to 99 h 22 min in 2023.

### HCRU for CAR T-cell 2023 therapy compared with ASCT therapy

**Fig. 5:**
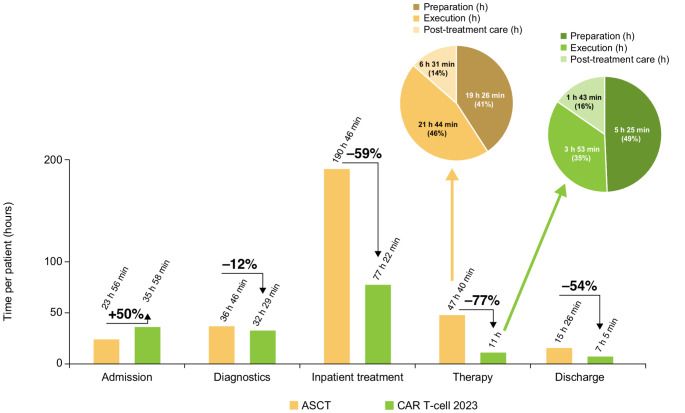
Personnel time by process category for the provision of CAR T-cell 2023 therapy compared with ASCT therapy. *ASCT* autologous stem cell transplantation, *CAR* chimeric antigen receptor

HCRU for CAR T-cell therapy was notably lower compared with ASCT therapy, primarily due to fewer treatment steps, the omission of salvage and high-dose chemotherapies, and shorter hospitalization. The average hospitalization for CAR T-cell therapy was 11 to 13 days, which was 31 to 33 days (70–75%) shorter compared with 44 days for ASCT therapy, including salvage and high-dose chemotherapy. Compared with ASCT therapy, the number of clinical pathway steps and underlying processes for the CAR T-cell therapy was nearly halved. There were 198 fewer (47%) steps and 883 fewer processes (47%). This reduction corresponded to a total decrease of 48% in personnel time dedicated to patient care, with CAR T-cell therapy requiring 163 h 53 min compared with 314 h 34 min for ASCT (Table 3). Due to the shorter hospitalization associated with CAR T-cell therapy, the time for inpatient treatment decreased by 59% to 77 h 22 min compared with 190 h 46 min for ASCT therapy (Fig. 5). Furthermore, the time required for therapy administration was reduced by 77% from 47 h 40 min for ASCT to 11 h for CAR T-cell therapy.

**Table 3:** Comparison of CAR T-cell 2023 therapy and ASCT therapy

	CAR T-cell 2023 including bridging therapy and lymphodepletion chemotherapy	ASCT and high-dose chemotherapy ± salvage therapy	Change (%)
**Hospitalization days**	11–13	44 (24–25 when excluding salvage chemotherapy)	70–75 (48–54 when excluding salvage chemotherapy)
**Clinical therapy steps (i.e.**,** clinical services)**	226	424	47
**Underlaying processes**	991	1874	47
**Total personnel time for patient case and services provision**	163 h 53 min	314 h 34 min	48

*ASCT* autologous stem cell transplantation, *CAR* chimeric antigen receptor

**Fig. 6:**
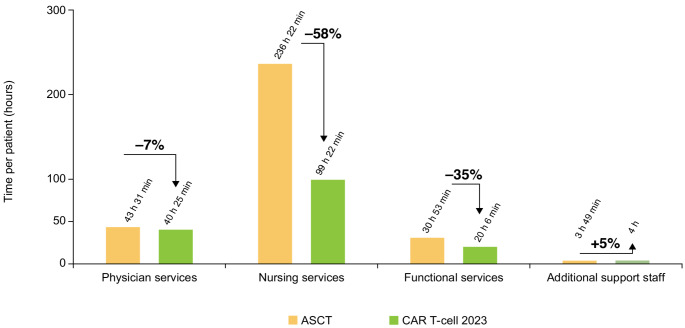
Total time by HCP group for the provision of CAR T-cell 2023 therapy compared with ASCT therapy. *ASCT* autologous stem cell transplantation, *CAR* chimeric antigen receptor, *HCP* healthcare professional

When assessing time utilization by HCPs (Fig. 6), the primary factors contributing to the overall difference between CAR T-cell therapy and ASCT were nursing and functional services. The time required for nursing services was 58% lower for CAR T-cell therapy, with 99 h 22 min compared with 236 h 22 min for ASCT. Similarly, functional support services were also reduced by 35%, with CAR T-cell therapy requiring 20 h 6 min compared with 30 h 53 min for ASCT therapy.

## Discussion

To the best of our knowledge, this study, conducted at Hospital St. Gallen, Switzerland is the first single-center process-orientated analysis addressing advancement of clinical practice in CAR T-cell therapy and its impact on HCRU. Treatment time, including hospitalization days, was shorter with the optimized CAR T-cell procedure in 2023 compared with 2022. A reduction in hospitalization of 30% was achieved by streamlining baseline laboratory and imaging assessments, and by moving lymphodepletion to the outpatient setting.

The combination of fludarabine and cyclophosphamide (FC) as lymphodepletion chemotherapy precedes the administration of the CAR T-cells. FC-containing regimens, with comparable doses and infusion times, are well-established outpatient treatment options for patients with chronic lymphocytic leukemia. According to our own clinical experience, FC is usually tolerated well by patients with a good overall performance status, which is also a precondition for CAR T-cell therapy. When discussing the different options of FC therapy administration, the majority of patients preferred the outpatient treatment to hospitalization, despite daily commuting. Patients clearly expressed a preference to reduce hospital admission prior to CAR T-cell administration and in the post–CAR T-cell monitoring phase.

Combined with optimization in frequency of assessments for CAR T-cell–specific toxicities and amendment of the procedure for the management of CRS and ICANS in the post–CAR T-cell infusion phase, a total personnel time reduction of around 15% from CAR T-cell 2022 therapy to CAR T-cell 2023 therapy was observed. Supportive of our findings, data from a systematic literature review that assessed the impact of outpatient versus inpatient administration of CAR T-cell therapies on safety, efficacy, quality of life, and HCRU outcomes in patients with hematologic cancer also showed lower overall HCRU in an outpatient versus an inpatient setting [22]. Moving the selected steps of CAR T-cell therapy to an outpatient setting liberates hospital resources, allowing for better allocation and reducing strain on inpatient facilities. Moreover, it is an important advancement in clinical practice that could provide patients with a more comfortable and convenient healthcare experience, helping to accommodate patient preferences [23], such as a shorter time in the hospital.

The introduction of CAR T-cell therapy, like any novel medical procedure, involves a necessary period of adaptation despite the rigorous regulations and a specific accreditation framework being in place [16, 24]. HCPs and centers involved in the provision of CAR T-cell therapy continually learn from experience, resulting in subsequent refinement of clinical processes. These process improvements can enhance the quality of patient care and optimize HCRU for the treatment center. Here we have documented the learning curve, starting from the initial introduction of CAR T-cell therapy where HCP experience is minimal, leading to proficient treatment of patients through learned experiences. These learnings can be used by other institutions that wish to introduce and/or refine CAR T-cell therapy.

In our study, the HCRU for the CAR T-cell therapy in 2023 was almost half that of the HCRU for the ASCT therapy. The therapeutic treatment administration processes were 77% lower with CAR T-cell 2023 therapy than with ASCT. As a reasonable explanation, ASCT therapy is often associated with patients experiencing bacteremia [25], sepsis [26], aplasia [27], or severe gastrointestinal mucositis [28] and requiring nutrition support or parenteral nutrition [29]; these therapy steps are less frequently needed in CAR T-cell therapy. During periods of physician and nursing shortages across Europe [30–32], an optimized CAR T-cell therapy pathway that reduces both physician and nursing time would be beneficial to healthcare systems.

The results of the present analysis are closely aligned with the findings of the comparable study conducted in Zurich [16], which also demonstrated a significant reduction in HCRU associated with the CAR T-cell procedure compared with ASCT. Both studies utilized the same methodology (ClipMed^PPM^) and observed that the most substantial decrease in treatment provision time occurred in nursing staff time and therapy administration workload—an important consideration given the widespread shortage of nursing personnel across Europe [30–32]. Both studies intentionally excluded management of severe AEs or ICU transfer and are comparable in this respect. However, in contrast to the Zurich study, the current study also mapped 9 follow-up visits over the approximately 2 months following patient discharge from the CAR-T procedure. Therefore, the total mapped days are higher for St. Gallen, i.e., 38 days in 2023 compared with 30 days in Zurich in 2020. Hospitalization days for CAR-T in 2022 at St. Gallen were slightly lower, at 18 days (if the full 10 days period post CAR-T administration has been accounted for), compared with 20 days in Zurich, which also included 10 hospitalization days post CAR-T infusion. Furthermore, the current study underscores the influence of the learning curve and the potential for continuous improvement adapting CAR T-cell treatment into routine clinical practice, noting a significant increase in efficiency over time. Relative to ASCT, a reduction of 48% in personnel time dedicated to patient care was observed in our study compared with a 31% reduction in the Zurich study [16].

This advancement highlights the potential of learning and provides concrete strategies for delivering resource-efficient, patient-centered, and future-oriented therapeutic interventions. These improvements, such as the movement of select processes from inpatient to outpatient, can be replicated in other centers. This enables the impactful changes resulting from our center’s learning to help other centers to expand CAR T-cell therapy and benefit a greater number of patients.

Strengths of our analysis include the well-established methodology, with prior use in over 300 studies. Limitations of our analysis are the single-center study design, which restricts the generalizability of findings due to site-specific SOPs or other national regulations, and the lack of patient and caregiver data collection, which did not allow any reduction in time and resources outside the healthcare system to be assessed, e.g., caregiver time. Overall treatment outcomes, costs, and cost effectiveness were not studied. Finally, ICU transfer and severe AE management were excluded from analysis to better represent and model the standard treatment pathway and was not intended to demonstrate all-exhaustive range of clinical experience or the real-world patient level data capturing. Further enhancement of this optimized CAR T-cell 2023 therapy delivery and AE management could explore any potential reduction of ICU transfer rates. Future multicenter studies may aid efficient CAR T-cell therapy integration across a diverse range of settings. Lastly, ClipMed^PPM^ uses a homogeneous patient population and follows a standardized procedure, which might not account for individual patient heterogeneity or severe complications of therapy. It also depends on the availability and quality of coded data and expert-based assumptions. Furthermore, the representation of “typical” care pathways is based on the synthesis of clinical experience and selected case reviews, which may introduce recall or selection bias.

In summary, the optimized CAR T-cell therapy provision in 2023 reduced HCRU by 15% compared with 2022, particularly reducing the burden on nurses by 21%. Moreover, by transitioning more of the steps to an outpatient setting, hospitalization could be reduced by 5 days (30%). These adaptations have led to more efficient use of healthcare resources, while at the same time potentially adding tangible value for patients. Such patient-centered value-based re-assessment of the treatment pathway that aims to align healthcare provision more closely with the actual needs and priorities of patients provides a foundation for sustainable improvement of medical care, healthcare resource use, and ultimately patient satisfaction. The optimization of our CAR-T processes is based on the experience gained through the evolution of the CAR-T procedure. This optimization is potentially applicable to any other CAR-T center, addressing hospital resources with limited capacity. These procedural improvements can be replicated in other centers, enhancing patient access to the innovative CAR T-cell therapy.

## Supplementary Information

Below is the link to the electronic supplementary material.

ESM 1 (PDF 257 KB)

## Data Availability

Primary data are available on request from Michael Greiling: michael.greiling@iwig-institut.de.
