# Supplementary material for: Advancement of clinical practice in delivering CAR T-cell therapy: impact on healthcare resource utilization and comparison with autologous stem cell transplantation in patients with relapsed/refractory large B-cell lymphomas
Source: Ann Hematol. 2025 Sep 6;104(9):4779–90. doi: 10.1007/s00277-025-06564-y (PMC12552251; doi:10.1007/s00277-025-06564-y)

Supplementary Information

Advancement of clinical practice in delivering CAR T-cell therapy: impact on healthcare resource utilization and comparison with autologous stem cell transplantation in patients with relapsed/refractory large B-cell lymphomas

Annals of Hematology

Martin Fehr[1] • Matthias Naegele[1] • Michael Greiling[2]

[1] Department of Medical Oncology and Haematology, Cantonal Hospital St. Gallen/HOCH Health Ostschweiz, St. Gallen, Switzerland

[2] Institute for Workflow-Management in Health Care, European University of Applied Sciences, Cologne, Germany

Corresponding author: Martin Fehr, Martin.Fehr@h-och.ch

Patient Selection

Information on the selection of patients, summary of internal guidelines, indication for treatment with commercially available CD19 chimeric antigen receptor (CAR) T-cell products, and clinical criteria as detailed in the respective checklist for submission to insurance companies when applying for reimbursement are included below.

Every case was discussed at the "*interdisciplinary haematological tumourboard"* for the confirmation of the indication. Treatments were indicated according to the registration of CD19 CAR T-cell therapy in Switzerland at that time: relapsed or refractory large B-cell non-Hodgkin lymphoma (LBCL) after 2 lines of therapy (including anthracyclines and CD20 antibody containing therapies). For reimbursement of commercially available CD19 CAR T-cell products, each patient was required to fulfill all of the following criteria:

- Age 18 years or older
- Adequate organ function with bilirubin < 2 mg/dl, aspartate aminotransferase (AST) and alanine transaminase (ALT) < 5-fold upper limit of normal, cardiac function with a left ventricular ejection fraction > 40% and no instable angina or myocardial infarction within the preceding 3 months
- Adequate hematological reserve defined as neutrophile granulocytes ≥ 1000/µl, platelets ≥ 50,000/µl
- No uncontrolled infections, in particular active hepatitis B, hepatitis C, and HIV
- No radiotherapy to the central nervous system during the previous 2 weeks, no new seizures, no new cerebral ischemia or cerebral hemorrhage, no dementia
- In case of preceding allogenic hematological stem cell transplantation: no active acute graft-versus-host disease (GVHD) grade 2–4, no extensive chronic GVHD, no donor

lymphocyte infusion (DLI) in the preceding 2 weeks, no T-cell–directed antibodies (e.g., alemtuzumab) in the preceding 8 weeks

- Eastern Cooperative Oncology Group (ECOG) performance status (PS) 0–1
- For female patients: no pregnancy, no breastfeeding

All the patients treated in our center fulfilled the respective criteria and the treatments were administered within routine clinical practice at the respective time.

Fig. S1 CAR T-cell 2023 therapy clinical pathway. *CAR* chimeric antigen receptor

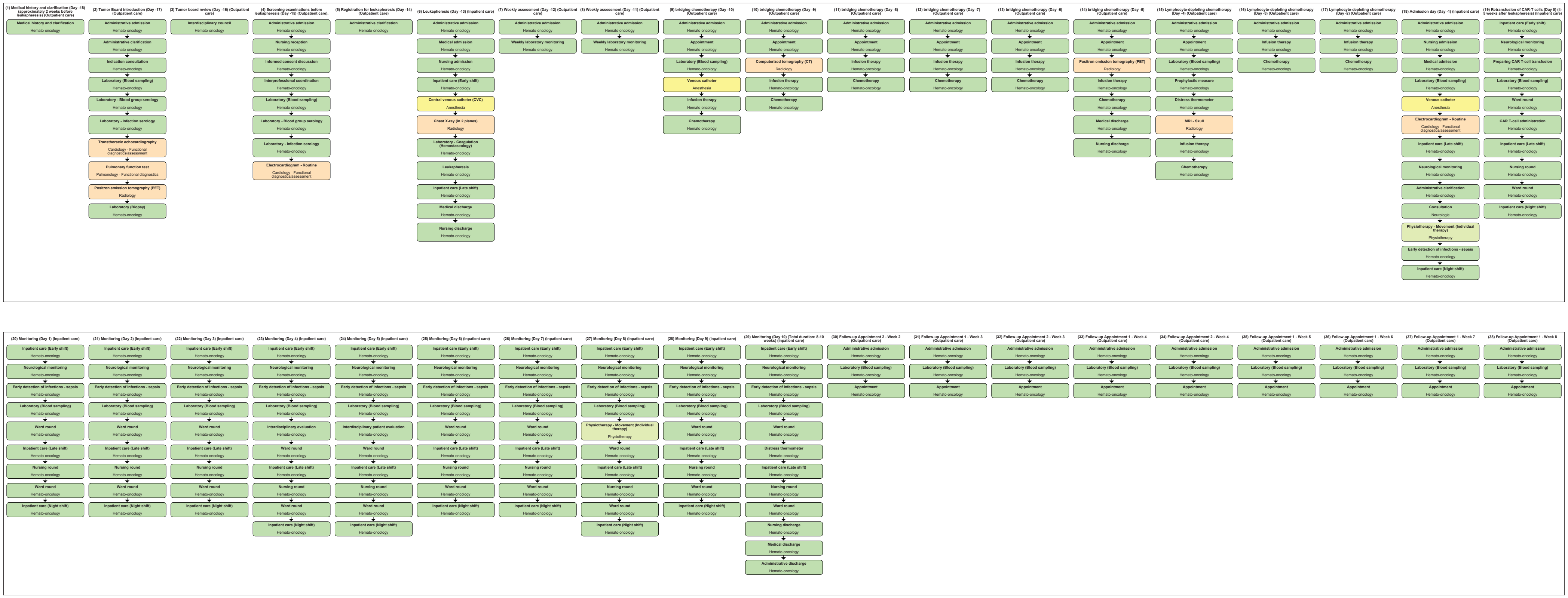

Fig. S2 ASCT therapy clinical pathway. *ASCT* autologous stem cell transplantation

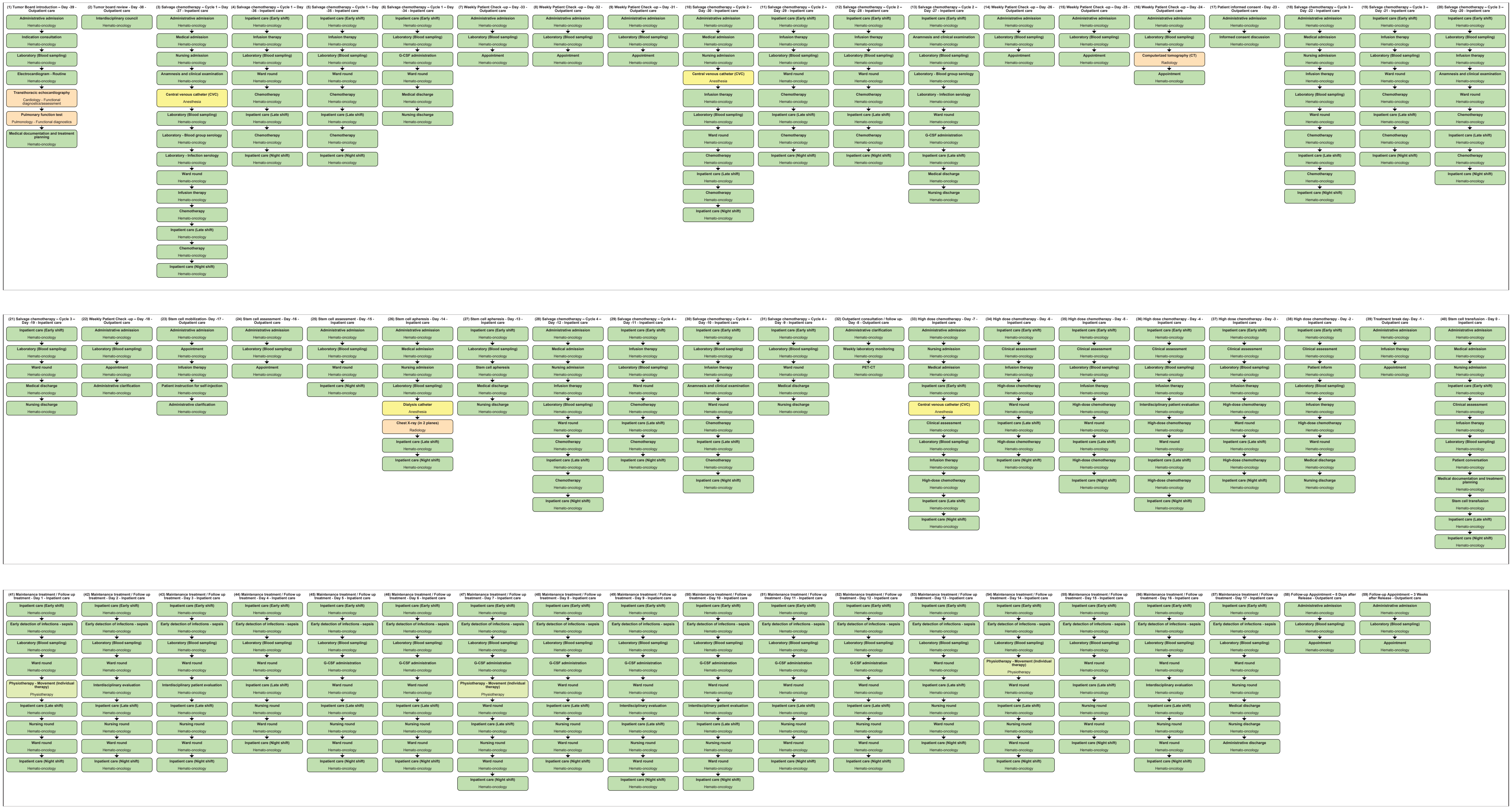

Supplement: Supplementary file 1 — (PDF 257 KB) [file 277_2025_6564_MOESM1_ESM.pdf]
